# Is orbital wall fracture associated with SARS-CoV-2 ocular surface contamination in asymptomatic COVID-19 patients?

**DOI:** 10.1007/s10792-022-02535-8

**Published:** 2022-09-24

**Authors:** Poramate Pitak-Arnnop, Chatpong Tangmanee, Jean-Paul Meningaud, Andreas Neff

**Affiliations:** 1 Faculty of Medicine, Philipps-University of Marburg, and Department of Oral and Craniomaxillofacial Plastic Surgery, UKGM GmbH, University Hospital of Marburg, Baldingerstraße, 35043 Marburg, Germany; 2 Department of Statistics, Chulalongkorn University Business School, Bangkok, Thailand; 3 Department of Plastic, Faculty of Medicine, Reconstructive, Esthetic and Maxillofacial Surgery, Henri Mondor University Hospital, AP-HPUniversity Paris-Est Créteil Val de Marne (Paris XII), Créteil, France

**Keywords:** SARS-CoV-2, Ocular surface, Contamination, Orbital fracture, Paranasal sinus

## Abstract

**Objectives:**

To assess the relationship between orbital wall fractures connecting to  paranasal sinuses (OWF-PNS) and SARS-CoV-2 ocular surface contamination (SARS-CoV-2-OSC) in asymptomatic COVID-19 patients.

**Methods:**

This was a prospective case–control study enrolling two asymptomatic COVID-19 patient cohorts with *vs.* without OWF-PNS in the case–control ratio of 1:4. All subjects were treated in a German level 1 trauma center during a one-year interval. The main predictor variable was the presence of OWF-PNS (case/control); cases with preoperative conjunctival positivity of SARS-CoV-2 were excluded to rule out the possibility of viral dissemination via the lacrimal gland and/or the nasolacrimal system. The main outcome variable was laboratory-confirmed SARS-CoV-2-OSC (yes/no). Descriptive and bivariate statistics were computed with a statistically significant *P* ≤ 0.05.

**Results:**

The samples comprised 11 cases and 44 controls (overall: 27.3% females; mean age, 52.7 ± 20.3 years [range, 19–85]). There was a significant association between OWF-PNS and SARS-CoV-2-OSC (*P* = 0.0001; odds ratio = 20.8; 95% confidence interval = 4.11–105.2; *R*-squared = 0.38; accuracy = 85.5%), regardless of orbital fracture location (orbital floor *vs.* medial wall versus both; *P* = 1.0).

**Conclusions:**

Asymptomatic COVID-19 patients with OWF-PNS are associated with a considerable and almost 21-fold increase in the risk of SARS-CoV-2-OSC, in comparison with those without facial fracture. This could suggest that OWF-PNS is the viral source, requiring particular attention during manipulation of ocular/orbital tissue to prevent viral transmission.

## Introduction

Recently, the present investigators performed a research series pertaining to COVID-19 and craniomaxillofacial surgery. One of our results is that ocular surfaces amid the nasal and oral cavities can potentially be the virus reservoir in COVID-19 patients [1, 2, 3, 4]. It remains unknown whether the orbital fracture could cause contamination of the ocular surface and/or orbital tissue with SARS-CoV-2. If so, the manipulation (i.e., examination and/or surgery) of ocular/orbital tissue in COVID-19 patients requires particular attention to prevent the viral transmission. Moreover, this viral presence was found to be linked to delayed wound healing and/or postoperative infection [2, 3, 4].

The purpose of this study was to answer the following clinical research question: “Among asymptomatic COVID-19 patients (i.e., orbital surgery in symptomatic COVID-19 patients was usually postponed), does orbital wall fracture exposing paranasal sinuses (OWF-PNS, i.e., proximity of maxillary and ethmoid sinuses to the orbital floor and medial orbital wall, respectively) increase the risk of SARS-CoV-2 ocular surface contamination (SARS-CoV-2-OSC)?” The null hypothesis was that there would no significant association between OWF-PNS and SARS-CoV-2-OSC. Our specific aims were to (1) measure SARS-CoV-2-OSC in patients with *vs.* without OWF-PNS, (2) estimate the association between OWF-PNS and SARS-CoV-2-OSC, and (3) discuss the possible relevance. We supposed the 2011 Oxford Centre for Evidence-Based Medicine’s Level of Evidence 3 at the study end, and the results would be the “first” case–control research findings on this matter.

## Materials and methods

### Study design/population

To address the research purpose, a prospective case–control study was designed and implemented. The study population was composed of asymptomatic COVID-19 patients with/without fractured midface (FM) treated in a German level 1 trauma center in a “hot-spot” COVID-19 area (> 65,000 severely infected case) during a one-year period.

Concerning inclusion criteria, “case” subjects were those with (1) age ≥ 18 years, (2) willing to participate in the study, (3) no evidence of underlying infections, e.g., rhinosinusitis, (4) asymptomatic COVID-19 (confirmed using the methods we previously explained [2, 3, 4]), (5) radiologically (via computed tomography [CT]) and intraoperatively confirmed OWF-PNS, and (6) at least one “emergent/urgent” indication for operative treatments, e.g., extensive open wound, retrobulbar hematoma, intracranial injury, or polytrauma [2]. The list of unrelated asymptomatic COVID-19 “control” subjects with “*no”* facial trauma (e.g., radius or hip fracture) was retrieved from the hospital’s patient database, and 4:1 matched by gender and age to individual “cases.” Exclusion criteria were subjects who (1) denied participation in the study, (2) had an underlying sinus and/or ocular/orbital disease such as chronic rhinosinusitis.

The institutional review board approved this study, and the Helsinki Declaration and the STROBE statement were followed throughout the study. All patients gave written consent to participate in this study and to allow the use of their anonymous data.

### Study variables

The predictor variable was the presence of OWF-PNS (yes/no) identified by the hospital’s database using the ICD (International Code of Disease), which reflected a complex combination of clinical and radiographic results for the definitive diagnosis. The outcome variable was RT-PCR-confirmed SARS-CoV-2-OSC (yes/no). The methods of RT-PCR for SARS-CoV-2 were described by Pitak-Arnnop et al*.* [2], and Atum et al*.* [6].

“Cases (with OWF-PNS)” were swabbed twice: (1) the first time was a preoperative conjunctival swab to rule out viral dissemination from the lacrimal gland and/or the nasolacrimal system and avoid the chance of intraoperative contamination, and (2) the second swab was performed intraoperatively at the OWF-PNS site directly and immediately when the fracture was exposed. In “control” subjects (without facial trauma), conjunctival swabbing was performed in the morning after they woke up because of probability of overnight microbial accumulation [5, 6, 7].

Other study variables were demographic parameters (age, gender).

### Data collection and statistical analysis

Anonymous patient data were converted into a collection form in Microsoft Excel 2007 (Microsoft Corp., Redmond, WA/USA). Using a statistical software MedCalc^®^ (MedCalc Software Ltd., Ostend/Belgium), descriptive statistics was computed for each of the study variables, and bivariate analyses were calculated to measure the association between the presence of OWF-PNS and RT-PCR-confirmed SARS-CoV-2-OSC. All evaluations were considered significant at *P* ≤ 0.05.

## Results

The study was conducted over 12 months including 55 patients (20% [or 11/55] had OWF-PNS; 80% [or 44/55] without facial fracture; 27.3% females) with a mean age of 52.7 ± 20.3 years (range, 19–85). Table 1 presents descriptive and bivariate analyses. SARS-CoV-2-OSC was found in 72% (or 8/11) and 11.4% (or 5/44; all reported multiple hand-eye contacts before the swab) of subjects with and without OWF-PNS, respectively (*P* = 0.0001; odds ratio = 20.8; 95% confidence interval [95% CI] = 4.11–105.2; *R*-squared = 0.38), regardless of the orbital fracture location (orbital floor *vs.* medial wall *vs.* both; *P* = 1.0).

**Table 1:** Cohort characteristics grouped by the presence of orbital wall fracture connecting with paranasal sinuses (OWF-PNS)

Parameters	Overall	Presence of OWF-PNS	Absence of OWF-PNS (no facial fracture)	*P* value (OR; 95% CI)
*Demographic*				
Sample size	55 (100)	11 (20)	44 (80)	N/A
Average age	52.7 ± 20.3 (19–85)	52.7 ± 21.8 (19–85)	52.8 ± 20.4 (20–83)	0.99 (N/A; − 14.04 to 13.95)
Age ≥ 55 years^§^	29 (52.7)	5 (17.2)	24 (82.7)	0.74 (0.69; 0.18 to 2.62)
Female gender	15 (27.3)	3 (20)	12 (80)	1.0 (1; 0.23 to 4.41)
*Outcome*				
Positive conjunctival swab RT-PCR	13 (23.6)	8 (61.5)	5 (38.5)	**0.0001** **(20.8; 4.11 to 105.2)**

^§^Median; RT-PCR—reverse transcription polymerase chain reaction; OR—odds ratio; 95% CI— 95% confidence interval; N/A — not applicable

Continuous data are listed as mean ± SD (range). Categorical data are presented as number (percentage). Statistically significant *P* values are indicated in bold typeface

With a prevalence of 0.236 (i.e., in this overall cohort of 55 patients, there were 13 patients with positive results from the conjunctival swab RT-PCR [or 23.6%]) and a probability of SARS-CoV-2-OSC cutoff point of 0.20 (i.e., it has generally been assumed and accepted that an adequate study power is 0.8 [80%], which would make β or chance of making a type II error to be 0.2 [20%]), the sensitivity, specificity, positive predictive value, negative predictive value and overall accuracy were 61.5%, 92.9%, 72.7%, 88.7%, and 85.5%, respectively. The secondary diagnostic test calculations demonstrated the posterior probabilities (odds) of positive and negative tests were 73% (95% CI, 45 to 90%) and 11% (95% CI, 6 to 20%). These results suggest that ~ 1 in every 1.4 patients with a positive test result is sick, and ~ 1 in every 1.1 with a negative test result is well (not infected).

## Discussion

Although early studies discarded SARS-CoV-2-OSC detection of [6, 7, 8, 9], conjunctival and corneal tissue connects with the upper respiratory tract [1, 2, 3, 4] and contains angiotensin-converting enzyme 2 receptors (ACE-2-R) for virus entry into host cells before replication of new virus particles that infect more cells [6, 7, 8, 9, 10, 11, 12]. Our previous studies revealed conjunctivitis in 6–32% of COVID-19 patients [1], and most orbital retractors were contaminated with SARS-CoV-2 during midfacial fracture repair [2]. Other systematic reviews elucidated low ocular-tissue tropism of this virus (range, 1–36%), which depends on patient’s symptoms [7, 8, 9, 10, 11, 12]. This finding is consistent with our results, i.e., 11.4% in non-OWF-PNS patients, or approximately 1 in every 10 asymptomatic COVID-19 patients without facial fracture will have SARS-CoV-2-OSC. Eye-hand contamination may need clinical concern.

Despite no control of confounding factors in this study, e.g., hand-eye contact time and frequency, effects of antimicrobial agents in tears (e.g., immunoglobulin A, lactoferrin, lysozyme, and lipocalin), blinking, or washing by tears [8, 11, 12], 61.5% of our “cases” had SARS-CoV-2-OSC. In other words, ocular surfaces of approximately 6 in every 10 asymptomatic COVID-19 patients with orbital fracture are contaminated with SARS-CoV-2. The possible explanation is a link between SARS-CoV-2-OSC and ACE-2-R in conjunctival epithelial cells and fibroblasts, corneal epithelial and endothelial cells, retina, and aqueous humor [7, 8, 9, 12]. The virus in the paranasal sinuses can sneak out through the fracture line (i.e., airborne contagion), herniated orbital fat, or blood clot [13, 14, 15] or could be inoculated in the lacrimal gland or its drainage system [8, 9, 11]. Albeit quarantined, the mouth and nostrils are also continuous droplet and aerosol generators in daily life [9, 12]. All of these paths may cause the re-infection of SARS-CoV-2 and/or prolonged viral shedding. Recently, we found extended SARS-CoV-2 shedding in nasal fracture patients, even though the swab tests were negative twice [4].

Another concern in COVID-19 patients is orbital infection. Apart from the commonly mentioned rhino-orbital-cerebral mucormycosis [16], COVID-19-related orbital abscess has been sporadically reported in the literature [17]. Indeed, approximately 1–6% of OWF (especially those with sinusitis 1–2 weeks pre-trauma or as long as 4–5 weeks post-injury [14, 15]) develop orbital cellulitis, and 4% of which extend intracranially, e.g., in the form of cavernous sinus thrombosis, meningitis, or subdural or cerebral empyema [13, 14, 15, 16, 17, 18, 19]. In our study, there was no orbital cellulitis because patients with underlying rhinosinusitis and/or eye diseases were excluded. However, anaerobic cellulitis resulting from orbital tissue hypoperfusion due to the fracture [16], or a COVID-prothrombotic event [1, 15, 17] may be developed in COVID-19 patients.

Weaknesses of this study are a small cohort, and unknown viral shedding dynamics which may decrease with time [7]. False negatives due to SARS-CoV-2 mutations, or poor collection, transport, or handling are also possible [11]. After reviewing the literature, we found that many other studies monotonously focused on facial trauma epidemiology during the pandemic [20, 21, 22, 23, 24]. To the best of our knowledge, this is the first case–control study concerning SARS-CoV-2-OSC in asymptomatic COVID-19 patients with OWF-PNS. Despite meager R-squared, the significant *P* and high overall accuracy suggest the good predictability of OWF-PNS on SARS-CoV-2-OSC. This research has, anyhow, thrown up many questions in need of further investigations, e.g., re-infection, surgical site infection, and viral transmission. Strict preventive measures should, therefore, be applied until proven otherwise.

## Conclusions

This study should be interpreted within the frame of the mentioned weaknesses. The results indicate that asymptomatic COVID-19 patients with any OWF-PNS locations (i.e., orbital floor, medial wall, or both) are almost 21 times more likely to have SARS-CoV-2-OSC than asymptomatic COVID-19 patients without the orbital/facial fracture. Although the case–control ratio and study design were likely to provide statistically relevant datasets, the present findings might not be representative of asymptomatic and/or symptomatic COVID-19 patients in other world regions. A large future study with more “cases” (which may be difficult because surgery for most of orbital fracture patients were postponed until the COVID-19 cures) will be beneficial in verifying or refuting the present findings. However, this work appears to be the very first look at the cause of SARS-CoV-2-OSC. OWF-PNS is the viral source and, thereby, should be manipulated cautiously.

## Data Availability

Deidentified individual participant data are not available. The datasets generated and analyzed during this study are available from the first author (P.P.) upon reasonable request.

## Abbreviations

ACE-2-R: Angiotensin-converting enzyme 2 receptors
95% CI: 95% confidence interval
COVID-19: Coronavirus disease 2019
CT: Computed tomography
FM: Fractured midface
OWF-PNS: Orbital wall fractures in close proximity to paranasal sinuses
RT-PCR: Reverse transcription polymerase chain reaction
SARS-CoV-2: Severe acute respiratory syndrome coronavirus type 2
SARS-CoV-2-OSC: SARS-CoV-2 ocular surface contamination
